# A Review on Marine Microbial Docosahexaenoic Acid Production Through Circular Economy, Fermentation Engineering, and Antioxidant Technology

**DOI:** 10.3390/md23060256

**Published:** 2025-06-16

**Authors:** Fengwei Yin, Xiaolong Sun, Xi Luo, Weilong Zheng, Longfei Yin, Yingying Zhang, Yongqian Fu

**Affiliations:** Taizhou Key Laboratory of Biomass Functional Materials Development and Application, School of Life Sciences, Taizhou University, Taizhou 318000, China; yinfengwei@126.com (F.Y.); sunxiaolong198901@163.com (X.S.); tzcluoxi@163.com (X.L.); z3037713@163.com (W.Z.); yinlongfei@163.com (L.Y.); zyying@tzc.edu.cn (Y.Z.)

**Keywords:** marine microbial DHA, circular economy, heterotrophic fermentation, oil quality enhancement, antioxidant technology

## Abstract

Marine microbial-derived docosahexaenoic acid (DHA) has garnered significant attention as a sustainable and health-promoting alternative to fish oil-derived DHA. However, its industrial production from marine heterotrophic microorganisms faces challenges related to high costs and suboptimal oil quality, which hinder its broader application. This review focuses on recent strategies aimed at achieving low-cost and high-quality marine microbial DHA production, emphasizing heterotrophic systems that dominate commercial supply. Key aspects include: Fermentation optimization using waste-derived feedstocks and bioprocess engineering to enhance DHA yields; Critical refining techniques—including degumming, neutralization, decolorization, and deodorization—are analyzed for improving DHA oil purity and quality, with emphasis on process optimization to adapt to the unique biochemical properties of microbial-derived oils. Additionally, strategies for oxidative stabilization, such as antioxidant protection, are discussed to extend the shelf life and preserve the nutritional value of marine microbial DHA oil. By integrating techno-economic and biochemical perspectives, this work outlines a holistic framework to guide the industrial optimization of marine microbial-sourced DHA oil production, addressing cost and quality challenges to facilitate its large-scale application as functional foods and nutraceuticals, thereby reducing reliance on marine resources and advancing sustainable omega-3 production.

## 1. Introduction

The increasing demand for healthier diets, fueled by rising living standards, is supported by scientific and technological advancements that enable material progress and meet evolving dietary requirements. Lipids, essential for human growth and development, encompass fatty acids that are classified as either saturated (SFAs) or unsaturated (UFAs). UFAs are further divided into monounsaturated fatty acids (MUFAs) and polyunsaturated fatty acids (PUFAs). PUFAs, owing to their unique chemical structure, possess a range of medicinal and health-promoting properties, such as anti-cardiovascular disease effects, cholesterol reduction, and the prevention of hypertension and diabetes. These properties have established PUFAs as integral to health-conscious dietary practices. Among PUFAs, ω-3 fatty acids (e.g., docosahexaenoic acid (DHA, C_22_H_32_O_2_) and eicosapentaenoic acid (EPA, C_20_H_30_O_2_)) and ω-6 fatty acids (e.g., gamma-linolenic acid (GLA, C_18_H_30_O_2_) and arachidonic acid (ARA, C_20_H_32_O_2_)) [1,2] are highly valued for their health benefits, including regulating anti-inflammatory pathways, supporting neurovascular health, and driving pro-inflammatory signaling and immune modulation, with their balanced interplay being essential for metabolic homeostasis and disease resilience [1]. Traditional sources of PUFAs, such as animal and plant oils, increasingly fall short of meeting growing demand. This shortfall has driven interest in microbial fermentation as an alternative production method, motivated by economic and societal considerations [3,4,5].

Docosahexaenoic acid (DHA, C_22_H_32_O_2_), an ω-3 long-chain polyunsaturated fatty acid, features six cis-configured double bonds. A colorless and odorless compound, DHA is insoluble in water but soluble in organic solvents and remains in an oily liquid state at room temperature due to its low melting point of −44 °C and inherent chemical instability. Despite its essential role in human metabolism, DHA cannot be synthesized endogenously and must be obtained from dietary sources. Often referred to as “brain gold”, DHA is crucial for brain and visual development, constituting a key component of neuronal cell membranes. Inadequate DHA intake during infancy can impair brain development and lead to cognitive delays, while sufficient levels in adults are vital for maintaining cognitive function [6,7,8].

Traditionally, DHA has been sourced from deep-sea fish and marine animals [9,10]. However, sustainable non-fish alternatives—collectively designated as “DHA algal oil” and primarily derived from heterotrophic marine microorganisms—are rapidly replacing conventional sources. Although derived from microbial fermentation, those DHA products are uniformly marketed as “algal oils” in accordance with global regulatory frameworks and consumer labeling conventions. Critically, this review specifically addresses high-yield DHA production from non-photosynthetic microbes, which constitute >90% of commercial algal DHA supply due to their superior productivity and fish-independent fermentation systems. These microbial platforms provide essential sustainability advantages, including decoupling from marine food webs and compatibility with circular bioeconomy models using waste feedstocks [11]. Despite its advantages, industrial-scale production of this microbial-sourced DHA (marketed as algal oil) faces significant challenges, primarily due to high production costs [10,12] and inconsistencies in product quality [13,14,15]. To address these issues, this review explores fermentation technologies and downstream processing methods aimed at cost reduction and quality control, highlighting the recent advancements in low-cost fermentation strategies, as well as refining and antioxidant technologies for marine microbial DHA. This integrated approach aims to enable affordable, high-quality DHA algal oil production at scale, reducing pressure on marine ecosystems while meeting growing nutraceutical demand.

## 2. Sources of DHA

### 2.1. Traditional Sources of DHA

Many DHA products on the market are labeled as “deep-sea fish oil”, reflecting the reliance on marine fish as the primary traditional source of DHA. Historically, fish from deep-sea regions, such as tuna, cod, herring, and sardines, have served as principal raw materials for DHA extraction [16]. However, in recent years, the use of fish oil as a DHA source has faced several challenges, primarily due to environmental and sustainability concerns, including global climate change and pollution [17,18].

Research indicates that DHA in seals, marine fish, and even commercial deep-sea fish oil consumed by indigenous populations, such as the Inuit, originates from DHA accumulation along the food chain. Planktonic microorganisms and microalgae in the ocean are capable of synthesizing DHA, with fish acquiring it by consuming these primary producers. Therefore, marine microbes and their phototrophic counterparts, rather than fish, are the true original producers of DHA [19].

### 2.2. New Sources of DHA

The limitations of traditional fish oil-derived DHA, coupled with the growing demand for high-quality DHA, have highlighted the insufficiency of fish oil to meet societal needs. As a result, there is an urgent need to identify high-quality, safe, reliable, and sustainable alternatives to fish-derived DHA [20,21]. In this context, oil-producing marine microorganisms—the original producers of DHA—have garnered significant global attention as a promising source of DHA.

An innovative approach to DHA production involves utilizing these marine microbial producers, particularly heterotrophic species, fermented under optimized conditions to accumulate lipids rich in DHA within their cells. This method bypasses the complexities associated with the bioaccumulation of DHA through the marine food chain, enabling the direct production of DHA from its microbial origin. Currently, commercially available microbial DHA oils are primarily derived from heterotrophic marine protists. Compared to fish oil-derived DHA, marine microbial DHA (commercially labeled as algal oil) offers several key advantages:

- High productivity in fermentation systems: Under controlled fermentation conditions, selected heterotrophic strains exhibit rapid growth and can accumulate oil at levels as high as 50% of their dry cell weight.
- Closed-tank bioprocessing: Fermentation occurs in sterile bioreactors with defined and uncontaminated culture media. This controlled environment produces DHA oil with fewer impurities, superior quality, and enhanced safety.
- Efficient extraction processes: Microbial fermentation DHA production integrates seamlessly with downstream processing. Following fermentation, the oil can be efficiently extracted and refined without the geographical and seasonal constraints associated with fish-derived DHA, ensuring consistent production quality.
- Utilization of agricultural by-products: These microorganisms can be cultured using low-cost agricultural by-products and waste materials, such as waste molasses and glycerol, as fermentation substrates. This approach not only reduces production costs but also valorizes waste, contributing to environmental sustainability by mitigating pollution from waste disposal.

Marine microbial DHA thus represents a highly promising alternative to traditional sources, with broad industrial application potential [21,22,23,24].

### 2.3. DHA-Producing Strains

The exploration of microbial sources as a source of DHA began in the late 1970s and early 1980s. During this time, scientists identified specific microalgal strains capable of accumulating substantial amounts of DHA, a fatty acid essential for human health, particularly for brain development. This groundbreaking discovery laid the foundation for subsequent research into these microorganisms as a sustainable and environmentally friendly DHA source. Initially, DHA-producing microalgae were cultivated using autotrophic growth in photobioreactors. However, this method was hampered by slow cell growth rates and low DHA yields, which posed challenges for efficient DHA oil production and complicated downstream processing. The 1980s marked a turning point with the introduction of heterotrophic culture techniques, which revolutionized DHA production. By supplementing the culture medium with carbon and nitrogen sources, heterotrophic cultivation facilitated faster cell growth, higher biomass accumulation, and increased oil content. This approach quickly became the dominant method for microbial DHA fermentation.

The first microalgal strain applied to the industrial production of DHA was *Crypthecodinium cohnii*, using heterotrophic culture methods, and the resulting DHA oil was incorporated into infant formulas and health supplements as a nutritional enhancer, establishing the safety and suitability of microalgal DHA for human consumption [25]. Despite its initial success, *Crypthecodinium cohnii* faced significant limitations, including a slow growth rate, an extended fermentation period of approximately 400 h per batch, and relatively low DHA production efficiency. Consequently, efforts have been directed toward identifying more efficient DHA-producing strains. Thraustochytrids, as marine heterotrophic protists, have emerged as a promising group for DHA production [26]. Genera such as *Thraustochytrium* [27], *Schizochytrium* [28], and *Aurantiochytrium* [29] exhibit biotechnologically relevant DHA-producing capabilities, showing great potential for large-scale commercial production [30,31,32].

## 3. Marine Microbial DHA Fermentation

### 3.1. DHA Synthesis Pathway

Under environmental stress such as nutrient limitations (e.g., nitrogen or phosphorus deficiency), oleaginous microorganisms shift toward lipid synthesis and accumulation. DHA synthesis primarily occurs through two pathways: the fatty acid synthase (FAS) pathway and the polyketide synthase (PKS) pathway [26,33]. Both utilize acetyl-CoA as a precursor and rely on NADPH for energy. The FAS pathway, dominant in most photoautotrophic species, generates fatty acids through cyclic reactions including condensation, reduction, dehydration, and a second reduction (a four-step cycle that sequentially extends and processes the fatty acyl chain). The PKS pathway, employed by heterotrophic marine protists like Thraustochytrids, is a multi-enzyme complex system. Mechanistically distinct from FAS, PKS introduces unsaturated bonds during fatty acyl chain elongation [34].

Therefore, the preference for either pathway is largely strain-specific and influenced by environmental and nutritional conditions. Photoautotrophic microalgae such as *Nannochloropsis* and *Chlorella* typically utilize the FAS pathway, in which unsaturated fatty acids are synthesized through a series of elongation and desaturation reactions of saturated fatty acids. This process is favored under nitrogen-limited conditions and sufficient oxygen availability, which activate desaturase enzymes and promote lipid accumulation [35,36]. In contrast, heterotrophic Thraustochytrids such as *Schizochytrium* and *Aurantiochytrium* predominantly use the PKS pathway, a multi-modular enzyme system that enables the direct formation of DHA without desaturation steps. This pathway is especially active under high carbon-to-nitrogen (C/N) ratios, low oxygen levels, and in the presence of abundant organic carbon sources such as glucose or glycerol [37,38]. Moreover, low cultivation temperatures have been shown to enhance DHA accumulation in PKS-dominant strains, likely by modulating enzyme activity and promoting the entry of relatively large amounts of substrates into the PKS pathway [39]. Overall, the dominance of the FAS or PKS pathway is not only species-dependent but also closely linked to cultivation mode, carbon and nitrogen availability, and oxygen conditions.

### 3.2. Materials for Microbial DHA Fermentation

In commercial microbial DHA fermentation using heterotrophic strains, the composition of nutrients in the culture medium plays a pivotal role in promoting rapid biomass growth and efficient oil accumulation. The consumption of substrates by the cells is directly linked to cellular activity, growth rates, and the rate of product synthesis.

#### 3.2.1. Traditional Carbon Sources

Carbon sources are essential for the growth and metabolism of microorganisms, serving as the primary energy supply and structural foundation for cellular functions. For DHA, a long-chain fatty acid with 22 carbon atoms, a continuous and efficient supply of carbon is crucial for its synthesis and accumulation. The choice of carbon source directly influences microbial biomass production and DHA oil yield.

Heterotrophic production systems utilize diverse carbon substrates for growth and oil accumulation. Glucose and glycerol are the dominant industrial carbon sources [40,41,42]. Glucose has been widely recognized as an effective carbon source for DHA production. For example, *Schizochytrium* sp. can efficiently ferment glucose and fructose for DHA synthesis, although it is unable to metabolize sucrose [43]. It is well known that glucose is particularly effective in promoting DHA accumulation during fermentation. Glycerol, another widely used substrate, has also demonstrated excellent potential for DHA production. Additionally, glycerol has been found to enhance the conversion of short-chain saturated fatty acids into unsaturated fatty acids during the oil accumulation phase, further boosting DHA synthesis [40,44].

In addition to traditional carbon sources, C2 compounds such as ethanol and acetic acid have been explored as substrates for DHA fermentation. Research indicates that ethanol and acetic acid are viable carbon sources for DHA production in *Schizochytrium* and other heterotrophic strains, offering alternative options for substrate utilization [41,45].

#### 3.2.2. Traditional Nitrogen Sources

Nitrogen is a vital component for microbial cell synthesis, serving as a building block for proteins, nucleic acids, and enzymes. The choice of nitrogen source significantly influences DHA production during fermentation, with both organic and inorganic nitrogen sources commonly employed.

Organic nitrogen sources such as yeast extract, corn steep liquor, monosodium glutamate (MSG), and peptone are frequently used in DHA production [46]. These sources often provide additional growth factors, including amino acids, vitamins, and fatty acids, which support cellular metabolism and catalysis [47]. For instance, MSG has been identified as an optimal nitrogen source for promoting both biomass accumulation and DHA synthesis [46]. Corn steep liquor, a cost-effective organic nitrogen source, has also demonstrated effectiveness in enhancing DHA production [48]. In comparative studies, MSG enhanced glucose consumption and biomass accumulation in *Schizochytrium* sp., whereas ammonium sulfate, an inorganic nitrogen source, favored oil accumulation [49].

Inorganic nitrogen sources such as ammonium sulfate, ammonia, and sodium nitrate are widely used due to their cost-effectiveness and fast cellular uptake [46,50]. Ammonium sulfate is particularly notable for its dual function: it not only serves as a nitrogen source but also helps regulate pH during fermentation by lowering the medium’s pH. Similarly, ammonia is often used for its dual role in providing nitrogen and maintaining a stable pH during fermentation, which enhances process efficiency [51,52].

Carbon and nitrogen sources are critical for optimizing DHA production during microbial fermentation. Traditional carbon and nitrogen sources are usually more expensive. By carefully selecting and balancing carbon and nitrogen sources, fermentation processes can be optimized for higher DHA yields and cost efficiency. Table 1 summarizes the traditional carbon and nitrogen sources and their effects in marine microbial DHA production.

#### 3.2.3. Non-Traditional Low-Cost Materials

Glucose currently serves as the primary carbon source for microbial DHA production, with yeast extract commonly acting as the organic nitrogen source. However, glucose represents one of the most expensive components in industrial fermentation media, often exceeding 60% of total fermentation costs in microbial fermentations when used as the primary carbon source [62]. Despite significant advancements in marine microbial DHA production, challenges remain in scaling up the process and achieving practical implementation. Compared to fish oil-derived DHA, fermentation-sourced DHA oil production costs remain higher, limiting market competitiveness [46,59,63]. Among the various factors contributing to this, the cost of fermentation raw materials is a crucial consideration. The use of low-cost substrates in fermentation systems often compromises DHA yields [48]. To overcome this challenge, further optimization of raw material processing methods and increased efficiency in cell utilization through fermentation regulation are required.

##### Cheap Carbon Sources

To reduce production costs, there has been growing interest in utilizing waste products and low-cost agricultural by-products as carbon sources for microbial DHA fermentation. This strategy not only helps recycle resources but also minimizes environmental waste while lowering the overall cost of DHA production.

Several agricultural by-products have been explored as viable carbon sources for DHA production. Coconut water, a significant waste product in Southeast Asia, contains sugars like glucose and fructose, which can support *Schizochytrium* fermentation for DHA production [64]. The techno-economic analysis by Anni’s research team revealed that glucose replacement with cassava-derived hydrolysate decreased overall DHA manufacturing expenses by 63%, demonstrating the viability of cheap lignocellulosic biomass utilization in microbial lipid production systems [63]. Nguyen et al. demonstrated that utilizing sugarcane bagasse hydrolysate as a carbon source achieved DHA productivity of 17.35% lipid content, with the carbon source cost dramatically reduced to USD 56.1 per ton compared to conventional glucose-based systems (USD 903.9 per ton), representing a 93.8% cost reduction in carbon substrate expenditure [65,66]. Similarly, sweet sorghum straw juice, when used at a 50% addition ratio, achieved DHA productivity comparable to pure glucose fermentation [67]. Cassava pulp has also been identified as an effective substitute for glucose, with a higher economic yield of DHA [63]. Corn syrup, another agricultural by-product, successfully supported *Aurantiochytrium* sp., yielding up to 20.1 g/L of DHA [68]. Additionally, bean dregs from tofu production have been used as a carbon source for *Schizochytrium*, yielding significant DHA production [69].

Crude glycerol, a by-product from the biodiesel industry, has been recognized as a promising carbon source for DHA fermentation. Studies have shown that crude glycerol can support *Schizochytrium* growth, resulting in high biomass and DHA yields [70]. It was reported that cultivation of *Schizochytrium* sp. S31 using crude glycerol achieved DHA productivity of 23.97% lipid content [71]. Since the cost of crude glycerol is only one-ninth that of glucose (USD 100 vs. 903.9 per ton) [66], the substrate cost analysis revealed that crude glycerol expenditure represented merely 11% of the equivalent glucose-based carbon source requirement, demonstrating an 89% cost reduction potential in bulk DHA production systems.

Molasses hydrolysates, derived from sugar production, have also been successfully used as an alternative to glucose, significantly lowering raw material costs [72,73]. Similarly, spruce hydrolysate, an industrial by-product, has demonstrated potential as a sustainable carbon source for DHA fermentation, demonstratings significantly lower production costs than pure glucose-based carbon sources, with substitution achieving an estimated 70% cost reduction [74]. Other waste materials, such as sugarcane top hydrolysates and food waste hydrolysates, have been explored for their low-cost potential, and algae residues after oil extraction have proven effective in stimulating biomass growth during DHA fermentation [75,76].

Using waste products and agricultural by-products as carbon sources for DHA fermentation presents an effective strategy to reduce production costs and address environmental concerns. These low-cost materials not only reduce the financial burden of DHA production but also promote sustainability by recycling waste into valuable products.

##### Cheap Nitrogen Sources

In addition to exploring low-cost carbon sources, researchers have focused on affordable organic nitrogen sources to further reduce production costs in marine microbial DHA fermentation.

Spent brewery yeast, a by-product of brewing, contains a high protein content (23.6%) and has been explored as a nitrogen source in DHA fermentation. The hydrolysate derived from spent brewery yeast has been successfully used as a substitute for yeast extract in cultivating *Aurantiochytrium* sp. for DHA production [77]. It was also demonstrated that spent brewery yeast is a promising, recyclable material for DHA fermentation [78]. Similarly, algae residues, which remain after oil extraction from microalgae, have shown potential as nitrogen sources. These residues, rich in protein, are commonly repurposed as livestock feed [79], but the non-lipid biomass left after oil extraction can also be utilized in microbial fermentation. It was found that enzymatically hydrolyzed residues from *Chlorella* after oil extraction were effective for cultivating *Chlorella vulgaris* [80]. Likewise, *Schizochytrium* sp. residues after oil extraction could serve as a nitrogen source in DHA fermentation. Strategic substitution of 80% conventional yeast extract (YE) with algal residue extract in DHA bioproduction achieved an 80% cost reduction in nitrogen source expenditure. Similarly, *Schizochytrium* sp. residues after lipid extraction demonstrate viability as an alternative nitrogen substrate for DHA biosynthesis. Implementation of algal-derived residue extract enabled substitution of 80% conventional yeast extract, achieving a proportional 80% cost reduction in nitrogen source [73]. A parallel investigation demonstrated that strategic recycling of cellular residues as nitrogen substrates achieved a 67.31% reduction in nitrogen source expenditure for DHA biosynthesis, concomitant with a 12.75% increase in DHA yield [81]. These strategies achieve dual industrial objectives by simultaneously addressing cost reduction and productivity enhancement in industrial applications.

Other organic nitrogen sources, such as rapeseed meal hydrolysates, which are rich in organic nitrogen, have also been identified as a promising nitrogen source for DHA fermentation, particularly in *Crypthecodinium* cohnii [72]. A techno-economic evaluation by Wang’s team revealed that valorization of tofu processing wastewater as both a nitrogen source and a culture medium not only boosted DHA output by 3.6-fold (361.54% increase) in *Thraustochytrid* fermentation but also established a low-cost nutrient supply model through complete elimination of traditional nitrogen substrate inputs [82].

Utilizing non-traditional cheap organic nitrogen sources offers a sustainable and cost-effective approach to reducing DHA production costs. Algae residues, in particular, represent a viable alternative to traditional yeast extract, providing an efficient way to recycle waste materials into valuable resources for fermentation processes. These alternatives help promote both economic and environmental sustainability in DHA production.

##### Wastewater

Wastewater refers to the liquid produced in various industrial, agricultural, and household activities that contains water-soluble substances, often rich in organic and inorganic components such as nutrients, nitrogen, phosphorus, and sugars. The recycling and utilization of wastewater have become essential strategies for resource conservation, cost reduction, and promoting sustainability, especially in microbial fermentation processes. The reuse of wastewater not only helps reduce waste emissions but also significantly lowers production costs [83,84].

After microbial fermentation, a significant amount of wastewater is often generated, containing inorganic salts that help maintain the osmotic pressure of the fermentation broth, as well as nutrient substances and growth-promoting factors [84]. For example, brewery wastewater, containing essential salts and nutrients, has been successfully used in DHA fermentation. Yamasaki et al. utilized brewery wastewater to achieve a DHA concentration of 3.4 g/L [85]. Similarly, Song et al. repurposed wastewater from *Mortierella alpina* fermentation to replace pure water in DHA production by *Aurantiochytrium*, obtaining a DHA yield of 28.7 g/L, comparable to that using pure water [86]. Additionally, tofu whey wastewater, a by-product of tofu production, has shown promise as a nitrogen source for DHA fermentation. Wang et al. cultured *Schizochytrium* sp. S31 using tofu whey wastewater, achieving significant biomass and DHA productivity [82]. A mixture of saline wastewater and tofu whey wastewater further enhanced biomass and DHA yields [87]. Tofu whey wastewater can even replace commercial nutrients, such as glucose and peptone, leading to a fourfold increase in DHA production [88].

Furthermore, dairy wastewater, such as mozzarella stretching water, has also been successfully applied in DHA fermentation, resulting in a DHA yield of 1.21 g/L [78]. Kitchen wastewater, typically rich in nitrogen, phosphorus, and carbohydrates, has been used for DHA fermentation by *Aurantiochytrium* sp., yielding 7.23 g/L of DHA [89].

The use of agricultural by-products, food processing by-products, fermentation wastewater, and waste residues as raw materials for DHA fermentation offers both environmental and economic benefits. This approach reduces production costs by replacing expensive commercial media, supports the commercialization of marine microbial-sourced DHA oil, and helps minimize waste and environmental pollution. By recycling these waste streams, resource conservation is promoted, and a more sustainable, circular economy in the biotechnological industry is fostered, enhancing DHA production efficiency and sustainability. Table 2 summarizes the non-traditional low-cost materials and their effects on marine microbial DHA production.

### 3.3. pH Control in DHA Fermentation Processes

In heterotrophic DHA fermentation, pH is a critical parameter that influences both cell growth and metabolism [42,96]. Production strains exhibit varying optimal pH values at different stages of growth and product accumulation. In some cases, the optimal pH for cell growth may differ from that required for target product formation. Several studies have shown that the optimal pH for product formation does not always coincide with the ideal pH for cell growth [97]. Moreover, pH fluctuations during fermentation, caused by substrate consumption (particularly nitrogen sources), can impact intracellular and extracellular ion balances and enzyme activity, further affecting overall productivity, and these fluctuations can impact the intracellular and extracellular ion balance, as well as the activity of enzymes involved in the process [98].

Given its importance, pH control is essential for achieving optimal DHA production. This can be accomplished by carefully designing the initial pH [99] or by maintaining a constant pH throughout the fermentation process [100]. Furthermore, different pH values could significantly affect cell morphology [42]. Therefore, carefully designing the pH has been widely studied to optimize DHA production. For instance, Zhao et al. demonstrated that maintaining an initial pH of 7.0 during the growth phase and then adjusting to pH 5.0 for the production phase significantly enhanced DHA content, achieving a maximum yield of 11.44 g/L [101]. Similarly, Yin et al. applied a two-stage pH strategy using ammonia and citric acid as pH regulators in *Schizochytrium* sp. fermentations. They found that a pH of 7.0 was optimal for cell growth, while a lower pH of 5.0 was favorable for DHA synthesis, resulting in a high DHA yield of 32.75 g/L [42].

Both single-phase and two-phase pH regulation strategies offer effective means of optimizing DHA production during microbial fermentation. Single-phase strategies are simpler to implement and can balance cell growth and product accumulation, whereas two-phase strategies provide greater flexibility to meet the distinct pH needs of different fermentation stages, often resulting in enhanced DHA yields. Selecting the appropriate pH strategy depends on the specific marine microbial strain, process requirements, and production goals.

### 3.4. Osmotic Control in DHA Fermentation Processes

Osmotic pressure is a key factor influencing cell growth and metabolism, especially for marine heterotrophic strains, as a stable culture environment is essential for optimal microbial performance [102]. Inorganic salts play a crucial role in regulating osmotic pressure and maintaining cellular integrity by serving as nutrients and enzyme activators. Excessive osmotic stress can disrupt cellular metabolism: hypertonic conditions may cause cell death due to water loss, while hypotonic conditions can lead to cell swelling and potential lysis [103]. For example, under hypertonic conditions, *Schizochytrium* sp. synthesizes compatible solutes like cyclohexanol and betaine to maintain metabolic functions [104], and *Chlamydomonas reinhardtii* forms irregular “ghost” cells under high osmotic pressure [105]. Additionally, mineral elements such as Na^+^, K^+^, Ca^2+^, and Cl^−^ often remain in the fermentation broth after DHA production, helping to stabilize osmotic pressure and support marine microbial growth [86].

Osmotic pressure also affects metabolite production, with some microorganisms accumulating specific metabolites under certain osmotic conditions. For instance, *Torulopsis glabrata* accumulates pyruvate under hypertonic conditions [106], while *Thraustochytrium* sp. shows a reduction in oil content without inorganic salts [107]. The stage of marine microbial growth and oil accumulation can also influence the effect of osmotic pressure. High osmotic conditions favor seed proliferation, while low osmotic conditions promote PUFA accumulation [108]. Strategies that manipulate osmotic pressure during fermentation, such as partial reuse of fermentation wastewater, can optimize biomass accumulation and DHA productivity [84]. Additionally, substrate concentration also plays a role in maintaining osmotic balance, with continuous feeding strategies helping to sustain microbial physiological conditions and improve DHA yields [102].

Osmotic pressure, influenced by inorganic salts and mineral elements, is essential for maintaining cell integrity and supporting microbial metabolism, especially during DHA fermentation. Adjusting osmotic pressure at different stages of marine microbial growth and oil accumulation can enhance DHA productivity, making osmotic regulation an important factor in optimizing fermentation processes.

### 3.5. Two-Stage Regulation Strategy for DHA Fermentation Processes

As discussed, microbial DHA oil synthesis occurs in distinct phases, with cell biomass proliferation and lipid accumulation taking place at different stages of fermentation [109,110]. During the initial stage, when nutrients are abundant, cells prioritize division and growth, leading to an increase in non-lipid biomass. As nutrients, particularly nitrogen sources, become depleted, the cells shift their metabolic focus. The carbon source is redirected toward lipid synthesis, initiating the oil accumulation phase. During this phase, cell proliferation halts, and lipid accumulation becomes the primary metabolic activity, with non-lipid biomass levels remaining relatively constant [111,112]. The asynchronous mechanism of biomass growth and lipid accumulation provides a theoretical basis for the development of a two-stage regulation strategy.

#### 3.5.1. Two-Stage Dissolved Oxygen Regulation

The two-stage dissolved oxygen regulation strategy is designed to optimize the growth and oil accumulation phases of microbial fermentation, addressing the high aerobic oxygen demand during the initial growth phase and the relatively anaerobic conditions needed for DHA accumulation in the later stages. Qu et al. used the oxygen transfer coefficient (KLa) as a benchmark for oxygen supply, maintaining KLa at 150.1 h^−1^ for the first 40 h of fermentation, then reducing it to 88.5 h^−1^ for the remainder of the process. This two-stage KLa strategy resulted in 43.83% and 63.88% higher biomass and DHA content, respectively, compared to constant KLa conditions [109]. Zhang et al. addressed the insufficient aeration in traditional shake flasks by introducing aeration membranes and adjusting the shaking table speed. This modification increased DHA production efficiency by 60% and shortened the fermentation cycle [113]. Zhao et al. designed various agitator combinations to enhance dissolved oxygen and mixing, and their computational fluid dynamics (CFD) simulations revealed that a configuration with straight-blade, arrow-blade, and flat-blade impellers was most effective for both cell growth and DHA accumulation [114]. Guo et al. developed a membrane-pore-material agitator to enhance the KLa value and gas content, incorporating a two-stage ventilation strategy to achieve higher biomass and DHA yields—87.34% and 83.77% higher than conventional reactors [111]. Additionally, optimizing bubble size in bubble column bioreactors could replicate the benefits of a two-stage oxygen supply strategy, improving both cell growth and DHA production [115].

#### 3.5.2. Two-Stage Temperature Regulation

Temperature plays a crucial role in cell growth and metabolism, especially in oil-accumulating microorganisms. While low temperatures promote the synthesis of polyunsaturated fatty acids (PUFAs) by maintaining cellular fluidity, they can also slow down enzymatic activity, which may reduce the rate of oil accumulation. Conversely, higher temperatures favor cell growth and biomass accumulation but may reduce PUFA synthesis [39,116]. Such as a temperature of 30 °C during the cell growth phase, followed by a reduction to 20 °C for the DHA accumulation phase, led to a DHA content of 52% of the total fatty acids [117].

#### 3.5.3. Two-Stage Substrate Regulation

Substrate selection is crucial for both cell growth and metabolic flux partitioning, with nitrogen limitation being a key factor in driving cellular metabolism toward lipid accumulation. A higher carbon-to-nitrogen (C/N) ratio in the culture medium is favorable for triacylglycerol biosynthesis in production phases. In a two-stage fermentation strategy, the first stage supports biomass growth without nutrient restrictions, while the second stage introduces nitrogen limitation to promote lipid accumulation. For example, Silvina et al. used a two-stage medium strategy for *Aurantiochytrium limacinum*, where the cells were first cultured in a medium optimized for biomass production, followed by transfer to a high C/N ratio medium (55:1) for lipid accumulation. This strategy resulted in a DHA productivity of 3.7 g/(L·day) [118]. Li et al. also explored the use of glucose and glycerol as carbon sources for DHA fermentation by *Schizochytrium* sp. and found that glucose supported biomass growth, while glycerol promoted DHA accumulation [40]. Based on this, they implemented a two-stage substrate fermentation strategy where glucose was used for biomass growth and glycerol for DHA synthesis, improving both production efficiency and reducing costs. In addition, Yin et al. applied this two-stage strategy using glucose for biomass accumulation in *Schizochytrium* sp., followed by hydrolyzed molasses for DHA production. This strategy increased the utilization of inexpensive molasses, enhancing DHA production efficiency while reducing raw material costs [73]. Furthermore, the two-stage feeding control strategy has also been successfully applied using alternative nitrogen sources, such as waste *Pichia pastoris*, in industrial DHA fermentation [119].

**Figure 1:**
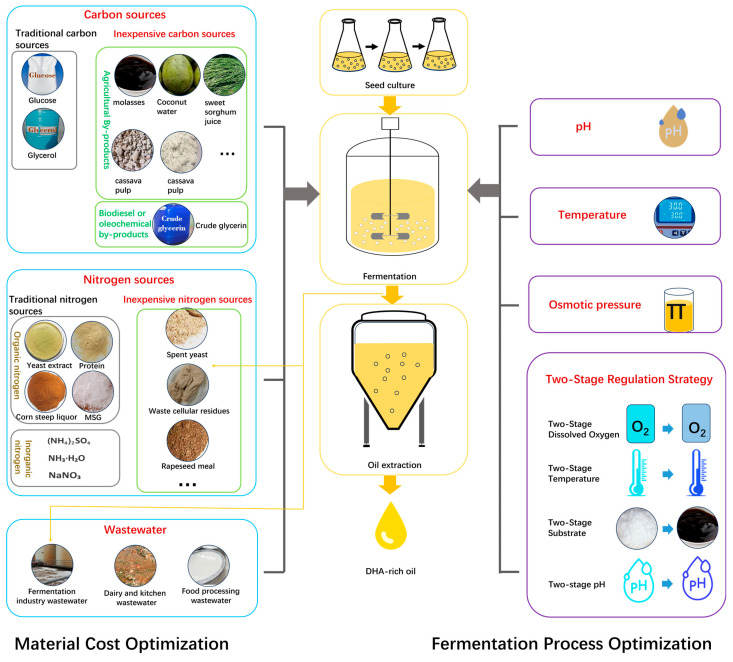
Schematic of marine microbial DHA fermentation: Cost-control and efficiency-enhancement strategies.

In summary, during marine microbial DHA fermentation, selecting suitable low-cost raw materials (such as agricultural waste or industrial by-products) to replace traditional expensive substrates can significantly reduce the economic cost of fermentation while promoting resource recycling and sustainable development. Meanwhile, optimizing key parameters in the fermentation process (such as temperature, pH, dissolved oxygen levels, and nutrient composition) and precisely controlling the fermentation conditions can enhance cell growth and DHA accumulation efficiency. This strategy provides essential technical support for achieving large-scale and cost-effective production of marine microbial-sourced DHA oil (Figure 1).

## 4. Post-Treatment of Marine Microbial DHA Oils

Marine microbial-sourced DHA oil exists both within the cell membrane as a functional lipid and in the cytoplasm as a storage lipid. To obtain DHA oil, several post-processing steps are necessary, including cell disruption and oil extraction. The resulting crude microbial oil contains various impurities such as pigments, proteins, and colloidal substances. Furthermore, during the extraction process, oxidation of certain lipids can lead to the formation of undesirable compounds, which can negatively impact the sensory qualities and fluidity of the DHA oil. Consequently, after the initial extraction, further refining treatments are required to remove these impurities and enhance the overall quality of the DHA oil.

### 4.1. Extraction of Oils

#### 4.1.1. Cell Lysis

DHA oil resides within microbial cells, necessitating cell disruption before oil extraction. Common methods for cell lysis include chemical, mechanical, and enzymatic approaches.

- Chemical lysis: This method uses strong acids or bases to break down the cell wall by dissolving glycoproteins, cellulose, and other structural components, thereby releasing the oil. While chemical lysis is relatively simple and does not require extensive sample pretreatment, the hot acid method is ineffective for extracting oils from cell membranes. Moreover, the use of acids and alkalis is highly corrosive and poses significant safety risks, making it unsuitable for large-scale production.
- Mechanical lysis: This technique utilizes high shear force from a high-pressure homogenizer to rupture cell walls and membrane components. Unlike chemical methods, mechanical lysis does not introduce chemicals, minimizing the risk of damaging the oil components and improving extraction efficiency. However, this method is energy-intensive and involves complex operational procedures, leading to higher labor and energy costs.
- Enzymatic lysis: This method uses specific enzymes tailored to the composition of the cell wall. The enzymes interact with the structural components of the cell wall and membrane, breaking them down and releasing the intracellular contents. Due to the complexity of the cell wall structures, a combination of enzymes is often required to achieve optimal lysis. With advancements in biotechnological enzymes and the increasing demand for efficient production methods, enzymatic lysis has become the predominant technique for disrupting DHA-producing microbial cells [120,121,122].

#### 4.1.2. Oils Extraction

Oil extraction from microbial biomass is commonly carried out using organic solvents due to the fat-soluble nature of oils. Organic solvents like n-hexane, cyclohexane, acetone, and chloroform are widely used for their ability to dissolve oils efficiently, as they are insoluble in water and have low boiling points that facilitate easy solvent removal through distillation [123,124]. However, organic solvent extraction has significant drawbacks. One of the primary concerns is the potential for solvent residues to remain in the extracted oil, which can affect oil quality and complicate the reuse of microbial residue. Furthermore, the flammability and volatility of organic solvents necessitate stringent safety measures during the extraction process, posing environmental and health risks to operators. These factors make the process less sustainable and raise concerns about its long-term viability.

To overcome these issues, supercritical fluid extraction, especially using supercritical carbon dioxide (CO_2_), has emerged as a promising alternative [125,126]. This method offers high purity and rapid separation, without the risk of solvent residues, making it an attractive choice for food-grade oil extraction [127,128]. Supercritical CO_2_ is particularly beneficial due to its ease of removal after extraction. However, the method’s sensitivity to the moisture content of the sample presents a challenge, as high-water content can hinder the extraction efficiency, requiring pre-drying of the biomass. Despite its advantages, supercritical fluid extraction is not yet widely adopted for marine microbial DHA extraction on an industrial scale. The high cost of equipment and operational complexities limit its application from laboratory production to industrial scale [124]. Consequently, researchers and manufacturers are increasingly exploring physical extraction methods, such as centrifugation, which offer safer and more efficient alternatives [83]. Marine microbial DHA-rich oil, composed of various fatty acids including palmitic acid (C16:0), docosapentaenoic acid (DPA, C22:5), and DHA, has a lower density than water due to the large molecular spacing between these fatty acids, making it well-suited for separation via centrifugation. The three-phase centrifuge is a particularly promising method for extracting microbial oil. It efficiently separates the oil, water, and solid phases into distinct layers, allowing for continuous and effective oil-water separation with minimal environmental impact, providing a safer, more sustainable extraction option [83,129]. The three-phase centrifuge enables continuous, high-efficiency DHA oil separation from microbial biomass, operating without interruptions to simultaneously isolate oil, nutrient-rich wastewater, and solid residues. The wastewater can be reused in fermentation media [83,84], while residues are repurposed as organic nitrogen sources [81]. This closed-loop process supports sustainable biomanufacturing by minimizing waste and maximizing resource recovery.

### 4.2. Oils Refining

Crude microbial DHA oil, after extraction, contains various impurities such as proteins, phospholipids, free fatty acids, and pigments, which can affect its safety, stability, and suitability for further processing. To improve the quality, appearance, and shelf life of the oil, refining processes are used to remove these contaminants. Oil refining typically involves several sequential steps, either carried out continuously or batchwise:

- Degumming: This step removes phospholipids and other gum-forming agents that can affect the oil’s clarity and quality.
- Neutralization: Free fatty acids are removed, which helps reduce acidity and improve the oil’s stability.
- Decolorization: Pigments and certain other contaminants are removed, improving the oil’s color and purity.
- Deodorization: Volatile compounds responsible for off-flavors and undesirable odors are eliminated to improve the oil’s sensory characteristics.

To enhance the oil’s fluidity and expand its potential applications in the healthcare and pharmaceutical industries, winterization is typically performed before deodorization. Winterization helps remove certain saturated fatty acids, ensuring the oil remains fluid even at lower temperatures.

#### 4.2.1. Degumming

Colloidal impurities in crude microbial DHA oils (phospholipids, proteins, glyceryl esters) compromise oil stability and complicate subsequent refining steps—such as causing emulsification during alkali refining or increasing decolorizing agent consumption. Thus, degumming acts as the first critical step in oil refining to address these issues [130].

The most common industrial degumming process involves adding salt water or phosphoric acid under heat: this hydrates and coagulates colloidal substances, which are then removed via sedimentation and separation. In recent years, more efficient technologies have emerged to overcome limitations of traditional methods. One is enzymatic degumming, which uses phospholipases to hydrolyze non-hydrated phospholipids in crude oil; released fatty acids enhance phospholipids’ hydrophilicity, enabling their transfer to the aqueous phase for separation, and this environmentally friendly approach also offers operational simplicity [131,132]. And several commercial phospholipases, such as Lecitase Ultra, GumZyme, and Lysomax, have been successfully developed and applied [130]. Another is ultrafiltration membrane separation: while phospholipids and glyceryl esters (with similar molecular weights of 700–900 Da) are difficult to separate with conventional membranes, phospholipids’ amphiphilic nature allows them to form reverse micelles (≥2000 Da) in oil-water mixtures—these micelles enable specialized membranes to achieve efficient phospholipid removal, with efficiency reaching up to 89% [133,134].

#### 4.2.2. Neutralization

Free fatty acids (FFAs) in crude microbial DHA oils increase acidity and trigger oxidative rancidity, generating harmful compounds and degrading oil quality. Thus, alkali refining acts as a critical deacidification step to address these issues.

The process involves adding alkaline agents (e.g., NaOH, KOH, Na_2_CO_3_, LiOH, Ca(OH)_2_, CaCO_3_, and NH_4_OH) to neutralize FFAs [134], with NaOH the most widely used. When NaOH reacts with FFAs, water-soluble sodium soaps form; these soaps are then removed by water washing, reducing acidity and the risk of rancidity. Precise alkali dosage is vital: insufficient alkali leaves residual FFAs (keeping the oil acidic), while excess alkali triggers saponification of neutral oils (increasing oil loss). Thus, accurate acid value determination is essential to calculate the optimal alkali amount. When properly controlled, alkali refining enhances oil stability, shelf life, and quality by neutralizing FFAs and inhibiting rancidity.

Beyond traditional alkaline methods, enzymatic deacidification offers an alternative [130], such as lipases (e.g., Novozym 435) selectively removing FFAs under mild conditions while minimizing DHA loss [135].

#### 4.2.3. Decolorization

Crude microbial DHA oil appears dark yellow after extraction, primarily due to pigments, notably carotenoids, the dominant pigments in marine microbial-sourced DHA [136]. These pigments degrade the oil’s appearance and functionality, so decolorization is essential to improve visual and functional quality.

Decolorization employs adsorbents (e.g., activated carbon, activated clay) to remove pigments, reduce color intensity, and eliminate impurities like metal ions and saponins. Choosing the right adsorbent is key to achieving effective decolorization [129,137]. Activated carbon (with a large surface area and micropores) effectively removes pigments but causes high oil loss due to strong oil adsorption. Activated clay (often derived from bentonite) offers high adsorptivity and chemical activity, enabling efficient decolorization with lower oil loss and filter residue. In some cases, combining multiple decolorants enhances pigment removal [138]. However, decolorization may deplete beneficial bioactives (e.g., vitamin E) and increase peroxide value [139]; thus, strict process control is vital to balance visual improvement with minimizing nutrient loss and preserving oil quality.

#### 4.2.4. Deodorization

Deodorization acts as the final critical step in oil refining, aiming to remove volatile odor-causing compounds (e.g., aldehydes, ketones, and free fatty acids) via steam distillation. This process not only eliminates unwanted flavors and odors but also enhances the oil’s stability, quality, and sensory properties [139]. Key parameters like temperature, pressure, duration, and stripping steam volume shape the process outcomes with inherent trade-offs: higher temperatures boost the removal of FFAs but increase the risk of trans fatty acid formation, which compromises the oil’s nutritional quality [140]; excessive stripping steam improves impurity removal yet leads to greater oil losses. For batch deodorization, typical conditions are 230–260 °C and 3–5 mbar, with the steam amount ranging from 5 to 15% of the oil’s weight. In continuous or semi-continuous systems, the steam volume is reduced to 0.5–2% of the oil’s weight to minimize losses [141].

The effectiveness of deodorization is often measured through the p-anisidine value, which quantifies secondary oxidation products like aldehydes and ketones [142]. Elevated levels of these compounds can adversely affect health by potentially raising blood pressure, interfering with the absorption of fat-soluble vitamins, and, in some cases, having carcinogenic properties. China’s DHA Algal Oil LS/T 3243-2015 requires the p-anisidine value to not exceed 15, and the industry often enforces even stricter standards. Case studies, like Yin et al.’s use of deoxygenated steam as a stripping gas, achieved a peroxide value of 0 meq/kg, a p-anisidine value of 3.50, and an acid value of 0.37 mg/g, producing DHA-rich oil that met high-quality market standards [15].

Beyond traditional steam distillation, innovations include using nitrogen as a stripping gas, which preserves heat-sensitive omega–3 fatty acids and reduces oxidative degradation [143], and nanofiltration membrane technology that can selectively remove volatile odorants under mild conditions, reducing thermal degradation compared to conventional high-temperature deodorization [135].

In summary, the post-treatment of marine microbial-sourced DHA oils is a critical chain for converting biomass into high-quality DHA-enriched products. Table 3 summarizes the refining processes and the specific impurities they target, visually illustrating the pathways for quality improvement. However, it should be noticed that most studies in this field currently prioritize technical performance indicators (e.g., extraction yield, purification efficiency, product quality) over economic evaluations across the entire post-treatment chain. Systematic cost data—which should cover raw material costs, equipment amortization, energy consumption, and other dimensions of multiple unit operations—are rarely reported in the literature. Without a unified quantitative model or validated data from industrial-scale practices, accurate cost calculations would remain speculative. Given this context, this section only focuses on the technical principles, key parameter optimization, and industrialization bottlenecks of each post-treatment unit (extraction and refining processes), aiming to provide a foundation for subsequent economic analyses and industrial-scale cost modeling in this domain.

## 5. Antioxidant Technology of Marine Microbial DHA Oils

### 5.1. Oxidation Processes in PUFA-Rich Oils

Oils enriched with polyunsaturated fatty acids (PUFAs) like DHA, are inherently vulnerable to oxidation during processing and storage. Exposure to light, heat, and oxygen triggers a series of oxidative reactions that not only reduce shelf life via rancidity development but also generate toxic by-products with potential health implications. The susceptibility of PUFAs to oxidation stems from the instability of their multiple double bonds, which render them highly reactive toward environmental stressors (e.g., light, heat, oxygen).

#### 5.1.1. Autoxidation of Oils

Autoxidation, a free-radical-mediated chain reaction, unfolds in three sequential stages: initiation, propagation, and termination.

Initiation commences with the abstraction of hydrogen atoms from unsaturated fatty acids or glycerides, yielding lipid radicals. Light, heat, and transition metals accelerate this step, with hydrogen atoms adjacent to double bonds (especially conjugated double bonds) being preferentially abstracted due to their lower bond energy.

Propagation stage: In this stage, lipid radicals react with molecular oxygen to form peroxyl radicals. These peroxyl radicals act as active chain carriers in the free radical chain reaction. They can attack new lipid molecules, leading to the formation of hydroperoxides and the generation of additional free radicals. The oxidation process continues as long as there is a source of hydrogen or until the chain reaction is interrupted by antioxidants or other inhibitors [144].

Termination neutralizes free radicals through radical–radical coupling or other interactions, forming stable non-radical products (e.g., free-radical polymers) and halting the chain reaction.

#### 5.1.2. Photo-Oxidation of Oils

Photo-oxidation diverges from autoxidation as it does not rely on free-radical initiation. Instead, triplet oxygen (in an excited state) directly abstracts hydrogen from unsaturated fatty acids, driving lipid oxidation. This process occurs via two mechanism-dependent pathways.

Type I (low oxygen concentration): Excited photosensitizers independently extract hydrogen from fatty acids, generating lipid free radicals analogous to those in autoxidation [145]. The excited photosensitizers then return to the ground state by reacting with these radicals, sustaining the oxidative process.

Type II (sufficient oxygen): Photosensitizers transfer energy to molecular oxygen, elevating it to the singlet state (^1^O_2_). This highly reactive ^1^O_2_ attacks electron-rich double bonds in unsaturated fatty acids at a rate ~1500-fold faster than ground-state oxygen, forming hydroperoxides [146].

### 5.2. Antioxidants for Marine Microbial DHA Oils

Oxidation not only shortens the shelf life of marine microbial-derived DHA-rich oils but also compromises their safety and nutritional quality by generating harmful by-products. Antioxidants are thus indispensable for inhibiting oxidation, extending shelf life, and preserving nutrient integrity. By definition, antioxidants interrupt oxidative chain reactions either by suppressing lipid free-radical formation or by reducing their concentration. Given DHA’s six unsaturated double bonds, hydrogen atoms adjacent to these bonds are particularly prone to abstraction, making DHA-rich oils exceptionally susceptible to oxidation [147] and necessitating robust antioxidant protection during storage.

Phenolic antioxidants, a prominent class of inhibitors, exert their effect by donating hydrogen atoms to lipid radicals, terminating the oxidative chain reaction. The resultant antioxidant radicals exhibit low reactivity and do not propagate new radicals; moreover, they can form stable non-radical products via interaction with lipid radicals, further quenching oxidation.

### 5.3. Types of Antioxidants

#### 5.3.1. Synthetic Antioxidants

Synthetic antioxidants are chemically engineered to inhibit oxidation at low concentrations (<200 ppm). Common examples include tert-butylhydroquinone (TBHQ), butylated hydroxytoluene (BHT), butylated hydroxyanisole (BHA), and ethylenediaminetetraacetic acid (EDTA).

TBHQ, a polar phenolic compound, demonstrates potent antioxidant activity—especially during the early stages of oxidation—and effectively extends the shelf life of DHA-rich fish oils, outperforming other synthetic analogs. EDTA, originally a chelating agent, mitigates oxidation by sequestering metal ions that catalyze hydroperoxide decomposition, thus preventing secondary oxidation. Despite their efficacy, synthetic antioxidants face scrutiny over potential health risks, driving consumer demand for natural alternatives [148].

#### 5.3.2. Natural Antioxidants

Natural antioxidants, abundant in plants, marine organisms, and microorganisms, protect host organisms from oxidative damage and offer applications in food preservation and nutraceuticals. Table 4 provides examples of natural antioxidants and their sources, highlighting the diversity of antioxidants found in nature and their broad potential applications.

- Tocopherol: A fat-soluble compound, tocopherol enhances DHA oil stability [149], though its efficacy is concentration-dependent. Structural isomers (α-, γ-, δ-tocopherols) exhibit varying activities; while tocopherol scavenges singlet oxygen and donates hydrogen to lipid radicals, excessive concentrations (>740 mg/kg) can paradoxically promote oxidation (e.g., by generating free radicals during decomposition) [150]).
- Rosemary extract: Derived from Rosmarinus officinalis, rosemary extract contains bioactive compounds (e.g., rosmarinic acid, carnosic acid, carnosol) and exists as water- or fat-soluble fractions. It is widely used in food preservation for its strong antioxidant properties [151,152], where a 50 mg/kg dose effectively controls oxidation rates [153], and it preserves the sensory quality of sardine oil during cold storage [154].
- Ascorbic acid: A water-soluble antioxidant, ascorbic acid neutralizes reactive oxygen species (ROS) via redox reactions [155] but exhibits limited solubility in oils. Esterification with fatty acids (e.g., ascorbyl palmitate) improves lipid compatibility while retaining antioxidant activity, making it suitable for DHA oil systems.
- Tea polyphenols: Extracted from tea leaves, tea polyphenols (e.g., catechins) are water-soluble and exhibit strong antioxidant activity. O’Sullivan et al. showed that 0.04% (*w*/*w*) tea polyphenols inhibit thermal degradation in frying oil [156]. Recent studies have further validated the antioxidant properties of tea polyphenols and demonstrated their successful application in protecting DHA algae oil [12,157,158].

### 5.4. Synergistic Effects of Antioxidants

While individual antioxidants target specific oxidation components or stages, their efficacy is often limited in isolation due to rapid activity loss. Combining antioxidants thus emerges as a strategic approach: synergistic interactions enhance overall antioxidant capacity, reduce dosage requirements, and lower costs [159]. These interactions amplify protective effects across multiple oxidative pathways and stages, providing comprehensive defense against oxidation [13].

#### 5.4.1. Vitamins and Polyphenols

Polyphenols exhibit amphiphilic properties, allowing them to dissolve in both water and oil, which enhances their ability to interact effectively with vitamin antioxidants in complex systems. Phenolic compounds with redox activity can also facilitate the regeneration of vitamins. For example, research by Dai et al. demonstrated that polyphenols could regenerate vitamin C [160]. In a model system using linoleic acid methyl ester peroxides, the flavonoid quercetin in catechins exhibited a synergistic effect with α-tocopherol by preventing chain oxidation of oils and regenerating α-tocopherol [161]. Specific bioactive compounds in rosemary extract (e.g., phenolic acids, diterpenoids), acting as hydrogen donors, can donate a hydrogen atom to the α-tocopherol radical, converting it back to its active form. Similarly, ascorbic acid can recycle oxidized α- and γ-tocopherol radicals back to their native state through direct reduction, thereby delaying tocopherol depletion [162]. The combination of green tea polyphenols (GTP), α-tocopherol, and ascorbate demonstrated cooperative inhibition of lipid peroxidation via a sequential redox cycling mechanism: GTPs reduced α-tocopheroxyl radicals to regenerate α-tocopherol, while ascorbate subsequently restored GTPs’ antioxidant capacity by neutralizing their oxidized derivatives [163].

A study on various phenolic compounds (e.g., chlorogenic acid, gallic acid, protocatechuic acid, and vanillic acid) in mango pulp revealed that most combinations, including all four compounds, exhibited significant synergistic effects [164]. Additionally, quercetin and resveratrol have also been proven to have a synergistic effect [165].

#### 5.4.2. Vitamins and Carotenoids

Carotenoids are effective quenchers of singlet oxygen, a highly reactive form of oxygen. They achieve this by absorbing excess energy and returning singlet oxygen to its ground state [147]. Marine microbial-sourced DHA oils naturally contains carotenoids, which are primary pigments contributing to the oil’s color [31,166]. Early studies have shown that β-carotene and α-tocopherol can offer mutual protection against the formation of linoleic acid peroxides, effectively preventing oxidation [167]. Carotenoids naturally present in palm oil could protect tocopherols from decomposition at high temperatures by undergoing their own oxidation. In turn, tocopherols can reduce some carotenoid free radicals back to their active forms [168]. However, carotenoids present a challenge in practical applications due to their dark color, which contradicts the goal of oil decolorization. As a result, carotenoids are typically removed during the decolorization process of DHA oil, limiting their use as antioxidants in this context.

#### 5.4.3. Application of Synergistic Antioxidants in DHA Oils

DHA oil, with its unique structure featuring six unsaturated double bonds, is highly susceptible to oxidation, influenced by both intrinsic and extrinsic factors. Therefore, selecting an appropriate antioxidant system is crucial to preserving the oil’s stability. Due to the synergistic effects and concentration dependencies among antioxidants, selecting the most effective antioxidant combination is not straightforward. Optimizing the combination and dosage often requires methodologies such as response surface methodology [157,169]. For instance, Shen et al. found that a combination of 80 mg/kg ascorbyl palmitate, 80 mg/kg vitamin E, 40 mg/kg phytic acid, and 80 mg/kg tea polyphenols provided better antioxidant protection than each antioxidant used individually, extending the shelf life of DHA-rich algal oils from 7.5 to 28.2 days [13]. Similarly, a combination of 53.20 mg/kg octyl gallate and 360 mg/kg tea polyphenol palmitate resulted in the highest antioxidant capacity, extending the shelf life of DHA algae oil by a factor of 4.24 [170]. In another study, Yin et al. optimized an antioxidant combination consisting of 0.0259% rosemary extract, 0.0224% vitamin E, and 0.0166% ascorbyl palmitate, which extended the oxidation induction time of marine microbial DHA-rich oils to 20.21–10.47 days longer than the control group—thus enhancing the antioxidant capacity and prolonging the shelf life of DHA oil [157]. While a study has suggested that the most effective protection for DHA algae oil involves four antioxidants, consisting of 80 mg/kg ascorbyl palmitate, 80 mg/kg vitamin E, 40 mg/kg phytic acid, and 80 mg/kg tea polyphenols [13].

**Figure 2:**
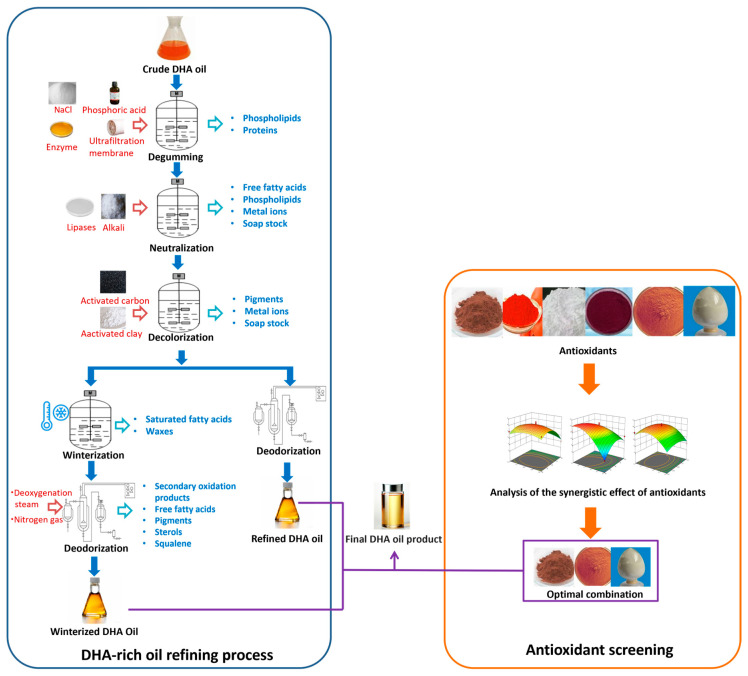
Quality enhancement framework for marine microbial DHA oil: Refining workflows and antioxidant selection.

In summary, crude marine microbial DHA oil obtained after oil extraction can undergo a series of refining processes, such as degumming, neutralization, decolorization, and deodorization, to remove impurities and achieve high-purity, high-quality DHA oil. Furthermore, the refined oil can be enhanced by adding appropriate antioxidants, such as vitamin E, polyphenols, or natural plant extracts, to effectively increase the oil’s antioxidant activity, reduce oxidation, and thus extend its shelf life (Figure 2). These measures not only enhance the quality of marine microbial-sourced DHA oil, but also broaden its applications across diverse sectors, including food and nutritional fortification, pharmaceuticals and healthcare, and animal feed and aquaculture industries. This progress aligns with the market demand for high-quality, stable DHA oils with extended shelf life.

## 6. Conclusions

The industrial production of marine microbial DHA oil has achieved significant advancements, particularly in enhancing production efficiency and improving oil quality. The shift toward utilizing heterotrophic microorganisms as a primary source of DHA presents a promising avenue for reducing production costs, primarily through the adoption of low-cost substrates and the optimization of fermentation processes. These developments not only lower the economic barriers to DHA production but also align with the broader goals of sustainable and environmentally friendly manufacturing practices. Ensuring the high quality of marine microbial-sourced DHA oil (commercially designated as algal oils) remains a critical priority, particularly in terms of its purity, stability, and nutritional value. Meeting these standards is essential for addressing market demands and maintaining consumer trust in the product. To this end, the implementation of robust quality preservation strategies, such as antioxidant treatments to mitigate oxidation, has proven effective. These measures are vital for extending the shelf life and maintaining the functional integrity of DHA oil, thereby enhancing its applicability in various industries, including food, pharmaceuticals, and nutraceuticals. Looking ahead, future research should focus on advancing the scalability of marine microbial DHA production processes to meet the escalating global demand for high-quality, cost-effective DHA oil. This includes the exploration of novel biotechnological approaches, such as genetic engineering and metabolic pathway optimization, to further enhance marine microbial productivity and DHA yield. Additionally, the development of innovative downstream processing techniques will be crucial for improving extraction efficiency and reducing energy consumption. Moreover, the integration of circular economy principles, such as the utilization of waste streams as substrates and the recycling of by-products, could further enhance the sustainability of marine microbial DHA production. Collaborative efforts among researchers, industry stakeholders, and policymakers will be essential to drive these innovations and ensure the widespread adoption of marine microbial DHA as a viable and sustainable alternative to traditional fish oil sources.

## Figures and Tables

**Table 1:** Conventional carbon and nitrogen sources for DHA production via fermentation.

Strains	Carbon Sources	Nitrogen Sources	Fermentation Conditions	DHA Content (g/L)	DHA Productivity (mg/L/h)	Ref.
*Thraustochytrium* sp.	Glucose	Yeast extract, peptone	Batch fermentation (shake flasks, 26 °C, 150 rpm, 120 h)	1.34	11.17	[53]
*Thraustochytrium* sp. ONC-T18	Glucose	Yeast extract, MSG	Batch fermentation (5 L bioreactor, 25 °C, 120 rpm, 168 h)	4.6	38.33	[54]
*Aurantiochytrium* sp.	Glucose	Yeast extract, peptone	Batch fermentation (shake flasks, 26 °C, 150 rpm, 120 h)	1.34	11.17	[55]
*Aurantiochytrium* sp. AF0043	Glucose, glycerol	MSG, Corn steep powder	Fed-batch fermentation (shake flasks, 28 °C, 150 rpm, 120 h)	2.75	22.92	[56]
*Aurantiochytrium* sp. PKU#SW8	Glucose	MSG	Batch fermentation (shake flasks, 28 °C, 170 rpm, 96 h)	3.64	37.92	[57]
*Aurantiochytrium* SW1	Fructose	MSG	Batch fermentation (shake flasks, 30 °C, 250 rpm, 120 h)	4.75	39.58	[58]
*Aurantiochytrium limacinum* SR21	Glucose, glycerol	Yeast extract, MSG	Fed-batch fermentation (5 L bioreactor, 25 °C, 300–400 rpm, 96 h)	32.36	337.1	[40]
*Schizochytrium* sp. I-F-9	Glucose, glycerol	Peptone, MSG	Fed-batch fermentation (shake flasks, 28 °C, 200 rpm, 120 h)	8.33	69.41	[38]
*Schizochytrium* sp. ATCC 20888	Glucose	Yeast extract, MSG	Batch fermentation (shake flasks, 25 °C, 200 rpm, 96 h)	6.95	72.4	[59]
*Schizochytrium* sp. HX-308	Glucose	Yeast extract, MSG	Three stage continuous fermentation (50 L bioreactor, 30 °C, 300 rpm, 147 h)	23.0	156.46	[60]
*Schizochytrium* sp. ABC101	Glucose	Yeast extract, corn steep liquor	Fed-batch fermentation (5 L bioreactor, 28 °C, 200 rpm, 84 h)	16.7	183.3	[61]

**Table 2:** DHA fermentation performance using alternative low-cost substrates.

Strains	Fermented Raw Materials	Biomass (g/L)	Lipid (g/L)	DHA (g/L)	Ref.
*Schizochytrium limacinum* SR21	Crude glycerol	7.89	4.94	1.84	[70]
*Schizochytrium limacinum* SR21	Sorghum straw sweat	9.38	6.90	2.35	[67]
*Schizochytrium limacinum* PA-968	Saline wastewater	28.40	9.82	3.1	[90]
*Schizochytrium mangrovei* Sk-02	Coconut wastewater	28.6	14.13	5.5	[64]
*Schizochytrium* sp. HX-308	Algal residues and cane molasses	78.26	35.54	15.22	[73]
*Schizochytrium limacinum OUC88*	Soybean meal hydrolysate	81.84	44.68	19.2	[91]
*Aurantiochytrium* sp. KRS101	Orange peel extract	5.5	2.85	0.78	[92]
*Aurantiochytrium* sp. KRS101	Spent yeast	31.8	12.12	10.4	[77]
*Aurantiochytrium* sp. SW1	Waste fruit extract	41.5	25.6	12.67	[93]
*Aurantiochytrium* sp. TZ209	Waste cellular residues	70.12	40.55	17.78	[81]
*Aurantiochytrium* sp. YLH70	Corn syrup	78.5	51	20.1	[68]
*Crypthecodinium cohnii* ATCC 30772	Crude glycerol	5.34	1.31	1.34	[94]
*Thraustochytrium* sp. (T18)	Lipid-extracted hydrolysate	14.86	6.43	2.07	[95]

**Table 3:** Oil refining processes and the removal of relevant impurities.

Oil Refining Processes	Major Impurity Components
Degumming	Phospholipids, proteins
Neutralization	Free fatty acids, phospholipids, metal ions, soap stock
Decolorization	Pigment, metal ions, and soap stock
Deodorization	Secondary oxidation products, free fatty acids, pigments, sterols, and squalene
Winterization	Saturated fatty acids

**Table 4:** Natural antioxidants and their sources.

Antioxidants	Examples	Sources
Tocopherol	α-, β-, γ-, δ-tocopherol	Seeds, grains, nuts, vegetable oils, etc.
Trienyltocopherol	α-, β-, γ-, δ- triene tocopherols	Palm oil, rice bran oil
Ascorbic acid	Vitamin C, ascorbate derivatives	Fruits, vegetables, etc.
Carotenoids	β-carotene, lycopene, lutein, astaxanthin	Carrots, tomatoes, microalgae, etc.
Phenols	Flavonoids, phenolic acids, tannins, lignans	Fruits, vegetables, grains, etc.
Peptides	Glutathione, metallothioneins, antioxidant peptides	Animal liver, eggs, milk, etc.
Enzymes	Superoxide dismutase, catalase, glutathione peroxidase	Plant and animal tissues

## Data Availability

Not applicable.
